# miR-149 inhibits cell proliferation and enhances chemosensitivity by targeting CDC42 and BCL2 in neuroblastoma

**DOI:** 10.1186/s12935-019-1082-9

**Published:** 2019-12-27

**Authors:** Fengxia Mao, Ju Zhang, Xinru Cheng, Qianya Xu

**Affiliations:** Department of Newborn Pediatrics, The First Affiliated Hospital of Zhengzhou University, No.1, East Jianshe Rd, Zhengzhou, 450052 China

**Keywords:** Neuroblastoma, miR-149, CDC42, BCL2, Proliferation, Chemosensitivity

## Abstract

**Background:**

Neuroblastoma (NB) is one of most common childhood tumors with high mortality among children worldwide. microRNAs (miRNAs) have been reported to play essential roles in the pathogenesis and therapeutics of NB. However, the role of miR-149 and its mechanism remain poorly understood.

**Main methods:**

The expression levels of miR-149, cell division cycle 42 (CDC42) and B-cell lymphoma 2 (BCL2) were measured in NB tissues or cells by quantitative real-time polymerase chain reaction or western blot. Cell proliferation was measured by 3-(4,5-dimethyl-2-thiazolyl)-2,5-diphenyl-2-*H*-tetrazolium bromide (MTT) and colony formation assays. Cell apoptosis was detected by flow cytometry. Chemosensitivity of NB cells to doxorubicin (Dox) was analyzed by MTT assay. The interaction between miR-149 and CDC42 or BCL2 was explored by luciferase activity and RNA immunoprecipitation analyses.

**Results:**

Our data indicated that low expression of miR-149 was displayed in NB tissues and cells and associated with poor survival rate. Overexpression of miR-149 inhibited cell proliferation and colony formation but promoted cell apoptosis and chemosensitivity to Dox in NB cells. Moreover, CDC42 and BCL2 were targeted by miR-149. Additionally, CDC42 and BCL2 mRNA levels were elevated in NB tissues and cells and restoration of CDC42 or BCL2 reversed the regulatory effect of miR-149 on NB progression.

**Conclusion:**

Our data suggested that miR-149 suppressed cell proliferation and improved Dox chemosensitivity by regulating CDC42 and BCL2 in NB, providing a novel avenue for treatment of NB.

## Background

Neuroblastoma (NB) is a common pediatric cancer wreaking havoc on health of children with high mortality worldwide [1]. Despite great advances in risk classification and treatment of NB, the survival of patients remains poor [2]. Emerging evidence indicates that proliferation and chemoresistance contribute to NB malignance [3]. Hence, exploring novel target for inhibition of development and chemoresistance may be promising for the treatment of NB.

microRNAs (miRNAs) are sing-stranded noncoding RNAs with 18-20 nucleotides in length, which could regulate post-transcriptional regulation of gene expression by binding with 3′-untranslated regions (3′-UTR) in NB [4]. Moreover, miRNAs are involved in the pathogenesis of NB by regulating development, chemoresistance and cancer stem cell function [5]. miR-149 is suggested to play an essential role in regulating cell proliferation, apoptosis, metastasis, chemoresistance and tumorigenesis in human cancers [6]. Accruing studies indicate that miR-149 might act as a tumor suppressor in various cancers, including gastric cancer, colorectal cancer, cervical cancer, nasopharyngeal carcinoma and ovarian cancer [7–11]. Notably, previous study reports that miR-149 is dysregulated in NB and inhibits NB cell proliferation by regulating Ras-related protein 1 [12]. However, the function and mechanism of miR-149 are complex, so more studies are needed.

Cell division cycle 42 (CDC42) is implicated in cell cycle progress, migration, invasion, growth and angiogenesis in multiple human cancers [13]. Furthermore, CDC42 is highly expressed and serves as an oncogene in many cancers, such as nasopharyngeal carcinoma, gastric cancer and glioma [14–16]. Additionally, previous work suggests that CDC42 is expressed in NB cells [17]. Meanwhile, apoptosis is regarded as a promising target therapy for various cancers [18]. B-cell lymphoma 2 (BCL2) is a key anti-apoptotic factor, which exhibits an important impact on the therapeutics of cancers [19]. Previous effort reports that miR-34a inhibits NB progression by suppressing BCL2 [20]. This also suggests that BCL2 is expressed and might act as an oncogene in NB. Interestingly, bioinformatics analysis provides the potential binding sites of miR-149 and CDC42 or BCL2, suggesting that CDC42 and BCL2 might be targeted via miR-149. However, there is no direct evidence in support of the relationship between miR-149 and CDC42 or BCL2 in NB. Thus, we hypothesized that CDC42 and BCL2 might be required for miR-149-mediated NB progression. In this study, we measured the expression levels of miR-149, CDC42 and BCL2 in NB. Moreover, we investigated the effect of miR-149 on cell proliferation, apoptosis as well as Dox chemosensitivity and explored the interaction between miR-149 and CDC42 or BCL2 in NB cells.

## Materials and methods

### Patients and tissue samples

A total of 42 patients with NB who had not received any chemotherapy and radiotherapy before surgery were recruited from the First Affiliated Hospital of Zhengzhou University. All participants have signed informed consent and the work was accepted by the Research Ethics Committee of the First Affiliated Hospital of Zhengzhou University. Paired tumor and normal samples collected from patients were immediately frozen in liquid nitrogen and then stored at − 80 °C until used. The clinical features of patients were displayed in Table 1. The 5-year survival rate was investigated after follow-up study.

**Table 1:** Association between NB patients’ clinicopathological factors and miR-149 expression

Clinicopathologic features	N (%)	Relative miR-149 level		*P* value
		High (%)	Low (%)	
Total cases	42 (100.0)	15 (35.7)	27 (64.3)	
Age (years)				0.3526
≥ 2.5	24 (57.1)	10 (41.7)	14 (58.3)	
< 2.5	18 (42.9)	5 (27.8)	13 (72.2)	
Gender				0.3939
Male	26 (61.9)	8 (30.8)	18 (69.2)	
Female	16 (38.1)	7 (43.8)	9 (56.2)	
INSS stage				0.0376
I–II	19 (45.2)	10 (52.6)	9 (47.4)	
III–IV	23 (54.8)	5 (21.7)	18 (78.3)	
Lymph node metastasis				0.0241
No	27 (64.3)	13 (48.1)	14 (51.9)	
Yes	15 (35.7)	2 (13.3)	13 (86.7)	

*NB* neuroblastoma, *INSS* International Neuroblastoma Staging System

### Cell culture and transfection

The human umbilical vein endothelial cells (HUVEC) and NB cell lines (SK-N-BE(2)C: MYCN amplified and TP53 variation at 404G>T; SK-N-SH: MYCN nonamplified and ALK variation at 3522C>A) were obtained from American Tissue Culture Collection (Manassas, VA, USA). All cells were cultured in Dulbecco’s Modified Eagle Medium (Gibco, Carlsbad, CA, USA) containing 10% fetal bovine serum (Gibco), 100 U/ml penicillin and 100 μg/ml streptomycin (Invitrogen, Carlsbad, CA, USA) at 37 °C in a humidified atmosphere with 5% CO_2_ during the study.

miR-149 mimic (miR-149) (sense: 5′-UCUGGCUCCGUGUCUUCACUCCC-3′; anti-sense: 5′-GGGAGUGAAGACACGGAGCCAGA-3′), miRNA negative control (miR-NC) (sense: 5′-UUCUCCGAACGUGUCACGUTT-3′; anti-sense: 5′-ACGUGACACGUUCGGAGAATT-3′), pcDNA, pcDNA-based CDC42 overexpression vector (CDC42) and BCL2 overexpression vector (BCL2) were synthesized by Genepharma (Shanghai, China). Cell transfection was conducted by using Lipofectamine 3000 (Invitrogen) according to the manufacturer’s instructions. Transfection efficacy was analyzed by quantitative real-time polymerase chain reaction (qRT-PCR) or western blot.

### qRT-PCR

Total RNA was isolated from tissues or cells by using TRIzol reagent (Invitrogen) following the manufacturer’s instructions. The complementary DNA (cDNA) was generated by using TaqMan microRNA Reverse Transcription Kit (Applied Biosystems, Foster City, CA, USA) or M-MLV Reverse Transcription Kit (Thermo Fisher, Wilmington, DE, USA), respectively, followed by amplification using SYBR green (Applied Biosystems) with the following amplification protocol: 95 °C for 5 min, 40 cycles of 95 °C for 15 s, and 60 °C for 1 min. Every sample was prepared in triplicate and the experiment was repeated three times. The expression levels of miR-149, CDC42 and BCL2 were calculated using 2^−ΔΔCt^ method with U6 small RNA or β-actin as endogenous control, respectively [21]. The primers were listed as follows: miR-149 (Forward, 5′-CATCCTTTCTGGCTCCGTGT-3′; Reverse, 5′-GCGTGATTCGTGCT CGTATATC-3′), U6 (Forward, 5′-CTCGCTTCGGCAGCACA-3′; Reverse, 5′-AACGCTTCACGAATTTGCGT-3′), CDC42 (Forward, 5′-CTTTCTTGCTTGTTGGGA CT-3′; Reverse, 5′-ACACCTGCGGCTCTTCTT-3′), BCL2 (Forward, 5′-CTGAGT ACCTGAACCGGCACC-3′; Reverse, 5′-GAGCAGAGTCTTCAGAGACAG-3′), β-actin (Forward, 5′-CAGCCTTCCTTCTTGGGTAT-3′; Reverse, 5′-TGGCATAG AGGTCTTTACGG-3′).

### Cell proliferation

3-(4,5-dimethyl-2-thiazolyl)-2,5-diphenyl-2-*H*-tetrazolium bromide (MTT) assay was performed to analyze proliferation of NB cells. After adjusting cell density to 5 × 10^4^/ml, 100 μl of SK-N-BE(2)C and SK-N-SH cells were seeded into 96-well plates and cultured for 0, 24, 48 or 72 h. Each group was prepared in triplicate. At the end points, cells were interacted with 0.5 mg/ml MTT solution (Thermo Fisher) for another 4 h, and then medium was replaced with 100 μl of dimethylsulfoxide (DMSO, Thermo Fisher) to dissolve formazan. The absorbance was measured at 490 nm using a microplate reader (Bio-Rad, Hercules, CA, USA).

### Colony formation assay

SK-N-BE(2)C and SK-N-SH cells were seeded into 6-well plates at a density of 500 cells per well. Following culture at 37 °C for 10 days, colonies were fixed with methanol (Sigma, St. Louis, MO, USA) for 30 min, and then stained with 0.01% crystal violet (Sigma) for 15 min. The colony formation was observed and counted under an inverted microscope (Olympus, Tokyo, Japan).

### Cell apoptosis

Flow cytometry was conducted to measure apoptosis of NB cells using Annexin V-fluorescein isothiocyanate (FITC)/propidium iodide (PI) apoptosis detection kit (Sigma) according to the manufacturer’s instructions. After incubation for 48 h, SK-N-BE(2)C and SK-N-SH cells were washed with PBS, resuspended in binding buffer and then double stained with Annexin V-FITC and PI. The apoptotic cells were analyzed by using a flow cytometer (Becton–Dickinson, Franklin Lakes, NJ, USA) with CellQuest software (BD Biosciences). Every sample was prepared in triplicate.

### Dox treatment and chemosensitivity assay

SK-N-BE(2)C and SK-N-SH cells were seeded in 96-well plates overnight. 0.2 μM Dox (Selleck, Shanghai, China) dissolved in DMSO in 100 μl of medium was added. After incubation for 48 h, cell proliferation, colony formation and apoptosis were measured as the above instructions. To investigate the Dox sensitivity, different concentrations (0–0.5 μM) of Dox were exposed to cells and the half maximal inhibitory concentration (IC50) was calculated through the growth curves.

### Luciferase activity assay

The putative binding sites of miR-149 and 3′-UTR sequences of CDC42 or BCL2 were predicted by starBase. pGL3 vectors (Promega, Madison, WI, USA) were used to synthesize wild-type (CDC42-WT, BCL2-WT) or mutant-type (CDC42-MUT, BCL2-MUT) luciferase reporter vectors containing wild-type or mutant binding sites of miR-149, respectively. Luciferase reporter vectors and miR-149 or miR-NC were co-transfected in SK-N-BE(2)C and SK-N-SH cells using Lipofectamine 3000 according to the manufacturer’s protocols. After the transfection for 48 h, luciferase activity was measured using luciferase assay kit (Promega) and normalized to Renilla luciferase activity.

### RNA immunoprecipitation (RIP)

RIP assay was performed in SK-N-BE(2)C and SK-N-SH cells by using RNA-binding protein immunoprecipitation kit (Millipore, Billerica, MA, USA) according to the manufacturer’s protocols. SK-N-BE(2)C and SK-N-SH cells transfected with miR-149 or miR-NC were lysed in RIP buffer with magnetic beads bound with Rabbit anti-Human antibody against Ago2 (anti-Ago2) (ab32381, 1:300 dilution), with IgG as a negative control. The mRNA levels of CDC42 and BCL2 enriched on beads were measured by qRT-PCR.

### Western blot

After washed with PBS, SK-N-BE(2)C and SK-N-SH cells were lysed in RIPA lysis buffer (Beyotime Biotechnology, Shanghai, China) containing 1% protease inhibitor (Beyotime Biotechnology). Total proteins were quantified by BCA protein assay kit (Beyotime Biotechnology) after centrifuged at 16,000×*g* for 20 min at 4 °C. Then proteins were denatured at 98 °C for 10 min, separated by SDS-PAGE and transferred to polyvinylidene difluoride membranes (Millipore). Subsequently, membranes were blocked with 5% non-fat milk in Tris-buffer saline containing 0.1% Tween 20 (TBST) for 1 h at room temperature, and then incubated with primary antibodies overnight at 4 °C and horseradish peroxidase (HRP)-conjugated secondary antibodies for 2 h at room temperature. The antibody against CDC42 (ab64533, 1:1000 dilution), BCL2 (ab59348, 1:500 dilution), β-actin (ab8227, 1:5000 dilution) and secondary antibodies (ab6721, 1:10,000 dilution) were purchased from Abcam (Cambridge, UK). β-actin was used as loading control in this study. The protein signals were analyzed with Image Lab software (Bio-Rad) after interacting with enhanced chemiluminescence (ECL) chromogenic substrate (Beyotime Biotechnology).

### Statistical analysis

The results were presented as the mean ± standard deviation (SD) from three independent experiments. The statistical differences between groups were analyzed by Student’s *t* test or one-way analysis of variance (ANOVA) followed by Tukey’s post hoc test using SPSS 18.0 software (SPSS, Inc., Chicago, IL, USA). Kaplan–Meier method was used to generate the survival curve of patients. Statistically significant was realized when *P* value was less than 0.05.

## Results

### miR-149 expression is reduced in NB

**Fig. 1:**
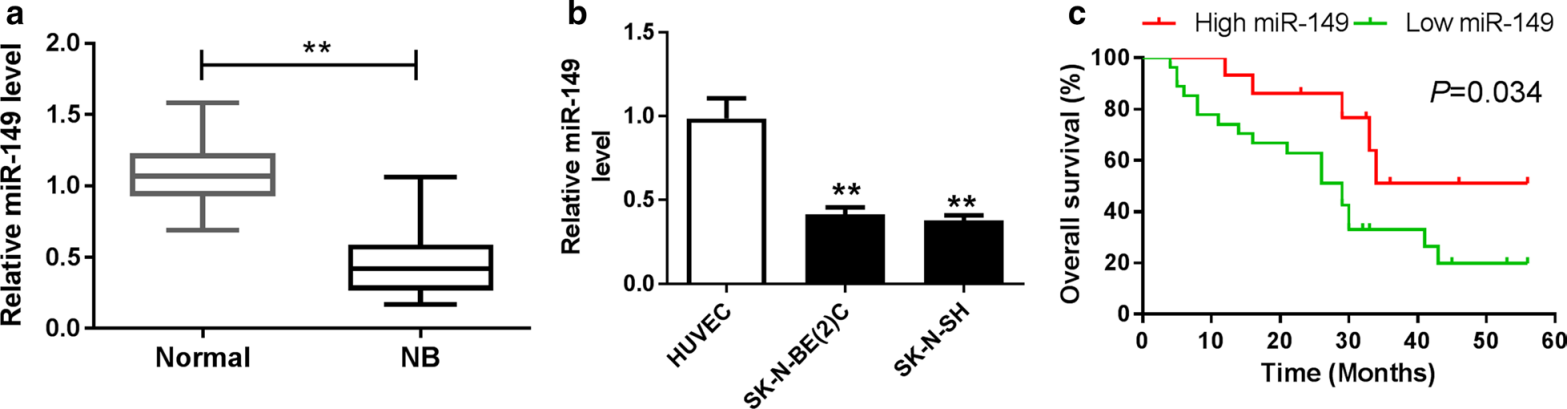
miR-149 expression was down-regulated in NB. **a** The expression of miR-149 was measured in NB tissues and normal adjacent samples by qRT-PCR. **b** The abundance of miR-149 was detected in NB cells and control HUVEC cells by qRT-PCR. **c** The overall survival was analyzed in patients with high or low expression of miR-149 by Kaplan–Meier method. **P < 0.01, compared with normal or HUVEC group

To explore the potential role of miR-149 in NB, its expression level was measured in NB tissues and cells. The expression of miR-149 was significantly reduced in NB tissues (n = 42) compared with that in normal samples (Fig. 1a). Similarly, SK-N-BE(2)C and SK-N-SH cells also displayed lower abundance of miR-149 than HUVEC cells (Fig. 1b). Moreover, the patients were classified as high miR-149 expression (n = 15) and low miR-149 expression (n = 27) according to the mean value of expression level. Table 1 and Fig. 1c summarized that low expression of miR-149 was associated with the International Neuroblastoma Staging System (INSS) stage (*P *= 0.0376), lymph node metastasis (*P *= 0.0241) and lower survival rate (*P *= 0.034) but not with age and gender of patients.

### Overexpression of miR-149 inhibits cell proliferation, colony formation while induces apoptosis in NB cells

**Fig. 2:**
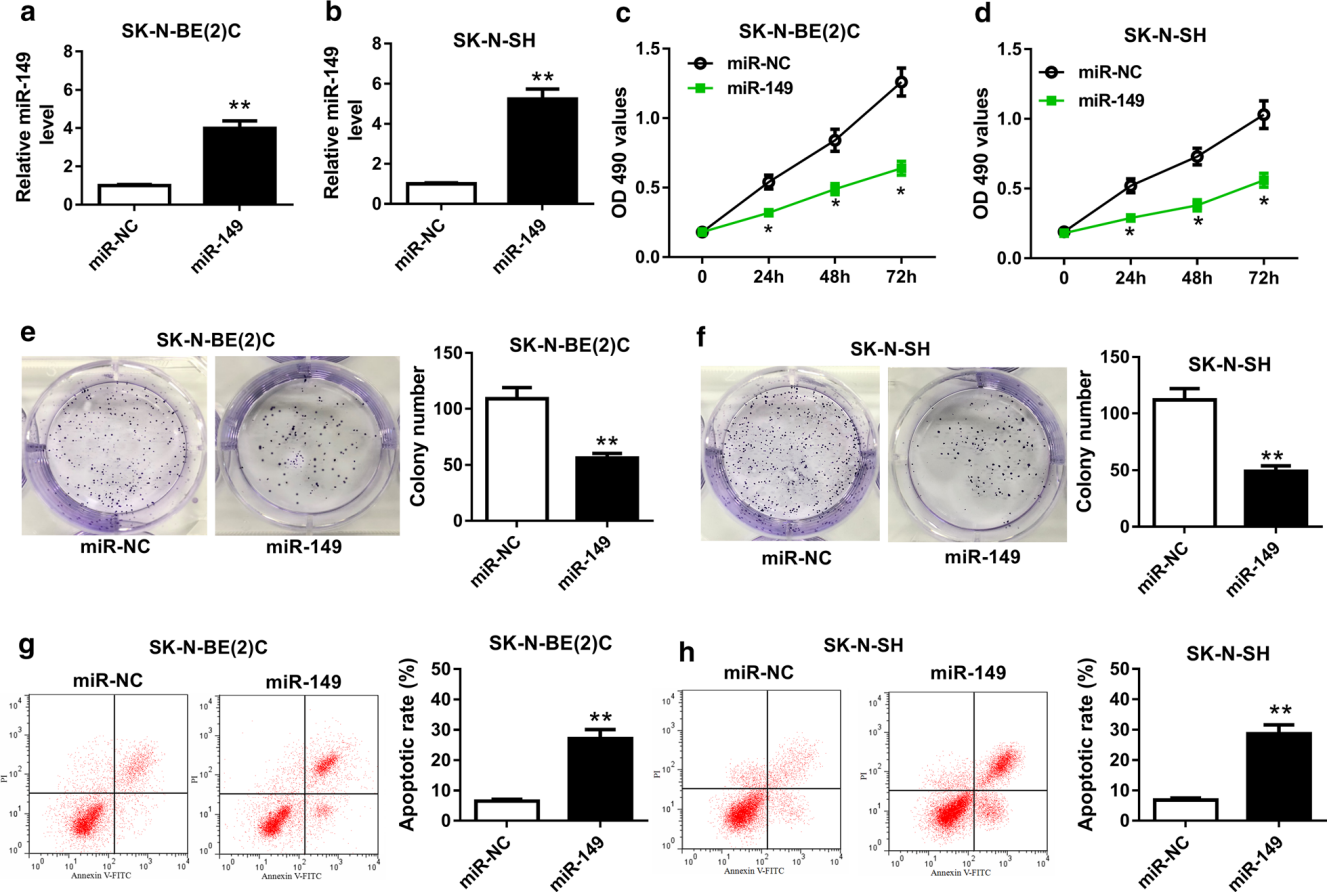
Overexpression of miR-149 inhibited cell proliferation, colony formation and promoted apoptosis in NB cells. **a**, **b** The expression of miR-149 was measured in SK-N-BE(2)C and SK-N-SH cells transfected with miR-149 or miR-NC by qRT-PCR. **c**, **d** Cell proliferation was detected in SK-N-BE(2)C and SK-N-SH cells transfected with miR-149 or miR-NC at different time points by MTT. **e**, **f** The numbers of colonies were analyzed in SK-N-BE(2)C and SK-N-SH cells transfected with miR-149 or miR-NC by colony formation assay. **g**, **h** Cell apoptosis was examined in SK-N-BE(2)C and SK-N-SH cells transfected with miR-149 or miR-NC by flow cytometry. *P < 0.05, **P < 0.01, compared with miR-NC group

To investigate the effect of miR-149 on NB progression, SK-N-BE(2)C and SK-N-SH cells were transfected with miR-149 or miR-NC. As a result, the abundance of miR-149 was effectively elevated in SK-N-BE(2)C and SK-N-SH cells after transfection of miR-149 compared with that in miR-NC group (Fig. 2a, b). MTT assay showed that addition of miR-149 led to great reduction of proliferation in SK-N-BE(2)C and SK-N-SH cells at 24, 48 or 72 h (Fig. 2c, d). Moreover, overexpression of miR-149 significantly impeded colony formation in SK-N-BE(2)C and SK-N-SH cells (Fig. 2e, f). However, the apoptotic rate was abnormally enhanced in SK-N-BE(2)C and SK-N-SH cells transfected with miR-149 compared with that in miR-NC group (Fig. 2g, h).

### Introduction of miR-149 facilitates Dox chemosensitivity to NB cells

**Fig. 3:**
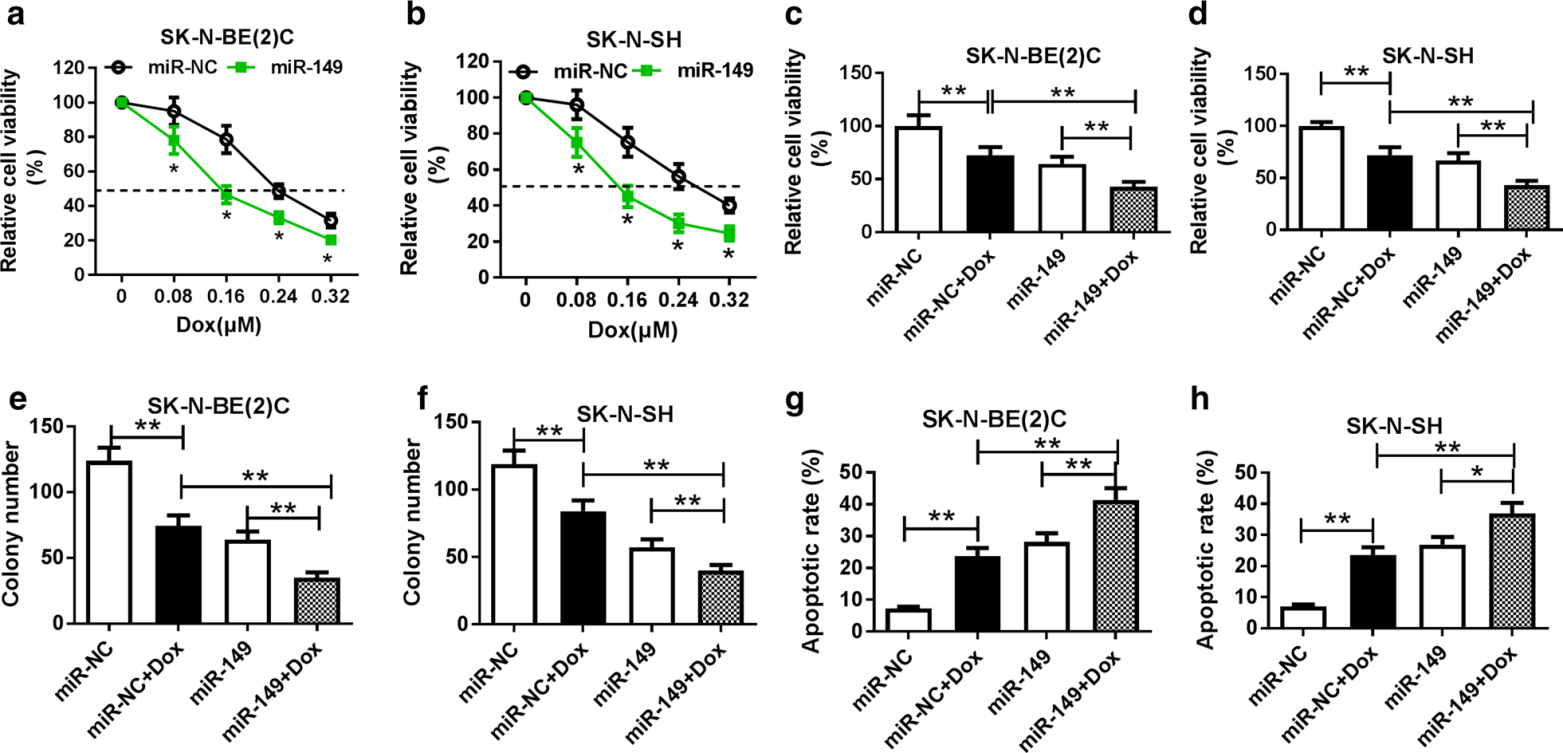
Addition of miR-149 contributed to sensitivity of NB cells to Dox. **a**–**d** Cell viability was analyzed in SK-N-BE(2)C and SK-N-SH cells transfected with miR-149 or miR-NC after treatment of Dox by MTT. **e**, **f** Colony formation was investigated in SK-N-BE(2)C and SK-N-SH cells transfected with miR-149 or miR-NC after treatment of Dox. **g**, **h** Cell apoptosis was measured in SK-N-BE(2)C and SK-N-SH cells transfected with miR-149 or miR-NC after treatment of Dox by flow cytometry. **P < 0.01, compared with non-Dox (miR-NC or miR-149) group

To evaluate the effect of miR-149 on chemosensitivity, SK-N-BE(2)C and SK-N-SH cells transfected with miR-149 or miR-NC were exposed to Dox for 48 h. After the exposure to Dox, cell proliferation was obviously decreased in SK-N-BE(2)C and SK-N-SH cells, and introduction of miR-149 exacerbated the inhibition of cell proliferation (Fig. 3a–d). Moreover, treatment of Dox lowered colony formation in SK-N-BE(2)C and SK-N-SH cells, which was aggravated by addition of miR-149 (Fig. 3e, f). Besides, the apoptotic rate was greatly enhanced by stimulation of Dox in SK-N-BE(2)C and SK-N-SH cells, and introduction of miR-149 deteriorated the effect (Fig. 3g, h). These findings suggested the promoting effect of miR-149 on Dox sensitivity to NB cells.

### CDC42 and BCL2 are targeted by miR-149

**Fig. 4:**
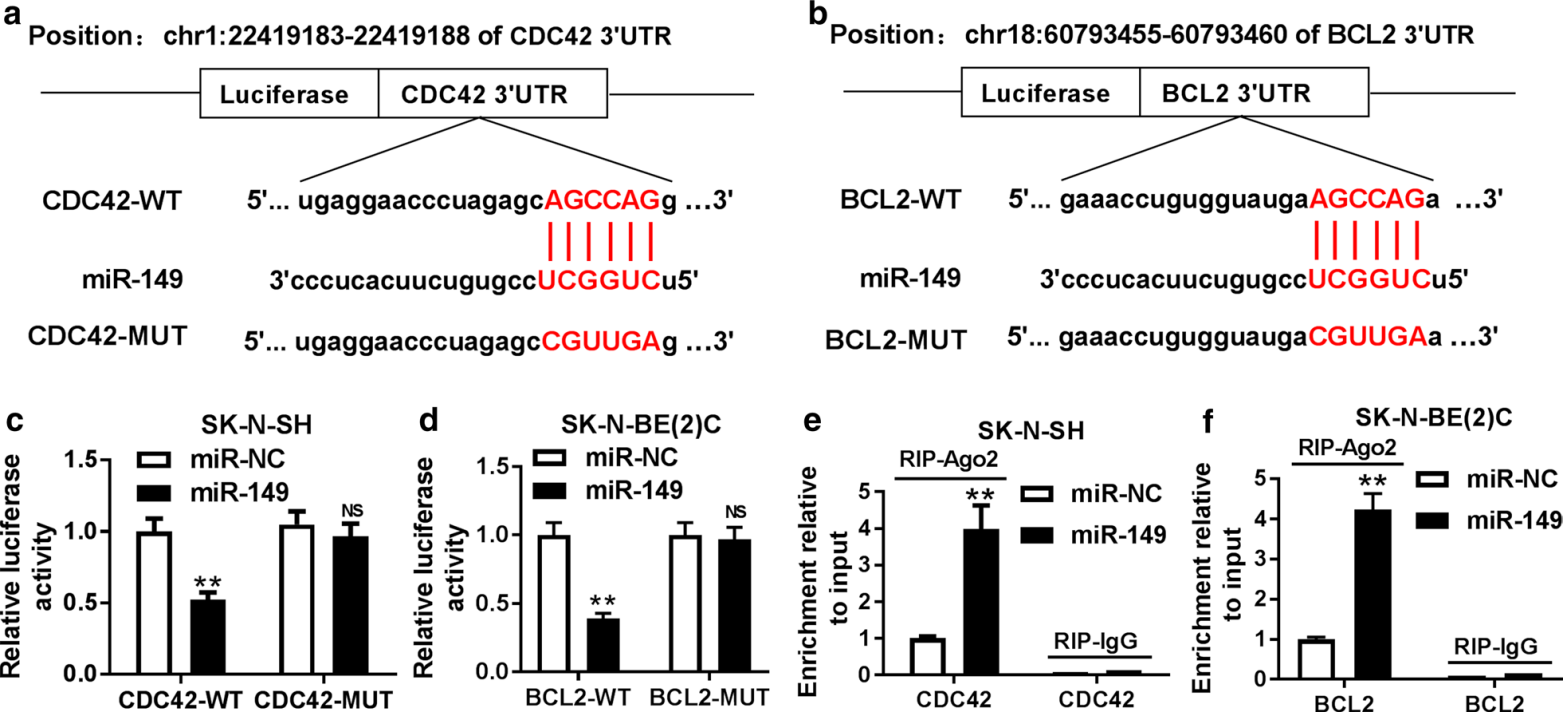
CDC42 and BCL2 were targeted by miR-149. **a**, **b** The putative binding sites of miR-149 and CDC42 or BCL2 were predicted by starBase. **c**, **d** Luciferase activity was analyzed in SK-N-SH and SK-N-BE(2)C cells co-transfected with luciferase reporter vectors and miR-149 or miR-NC. **e**, **f** The enrichment of CDC42 or BCL2 was detected in SK-N-SH and SK-N-BE(2)C cells transfected with miR-149 or miR-NC by RIP assay. NS: no significant, *P > 0.05; **P < 0.01, compared with miR-NC group

To explore the underlying mechanism allows miR-149 participating in NB, promising targets were needed to be probed in NB cells. Bioinformatics analysis using starBase showed, among 8 potential targets which were expressed in neuroblastoma, that CDC42 and BCL2 mRNA levels were up-regulated most in SK-N-SH cells (Additional file 1: Figure S1). Hence, these two were chosen for further experiments. The potential binding sites of miR-149 and CDC42 or BCL2 were shown in Fig. 4a, b, suggesting that CDC42 and BCL2 might be correlated with miR-149. Luciferase activity and RIP assays were widely used for validation of interaction between miRNA and target [22, 23]. In luciferase activity assay, 3′-UTR of targeted gene was cloned into the downstream of luciferase reporter gene; analyzing the effect of miRNA overexpression on luciferase activity implied the inhibitive role of miRNA in mRNA expression; combining with mutant constructs, the binding sites were further confirmed. Our results showed that overexpression of miR-149 resulted in a great loss of luciferase activity in SK-N-SH cells transfected with CDC42-WT, while its efficacy was lost in response to CDC42-MUT (Fig. 4c). Likewise, addition of miR-149 played similar role in luciferase activity in SK-N-BE(2)C cells transfected with BCL2-WT or BCL2-MUT (Fig. 4d). Moreover, up-regulation of miR-149 led to more enrichment of CDC42 and BCL2 by Ago2 RIP in SK-N-SH and SK-N-BE(2)C cells, whereas little efficacy of enrichment was shown in IgG RIP group (Fig. 4e, f). These results indicated that CDC42 and BCL2 could be targeted via miR-149 in NB cells.

### CDC42 and BCL2 mRNA levels are up-regulated in NB tissues and cells

**Fig. 5:**
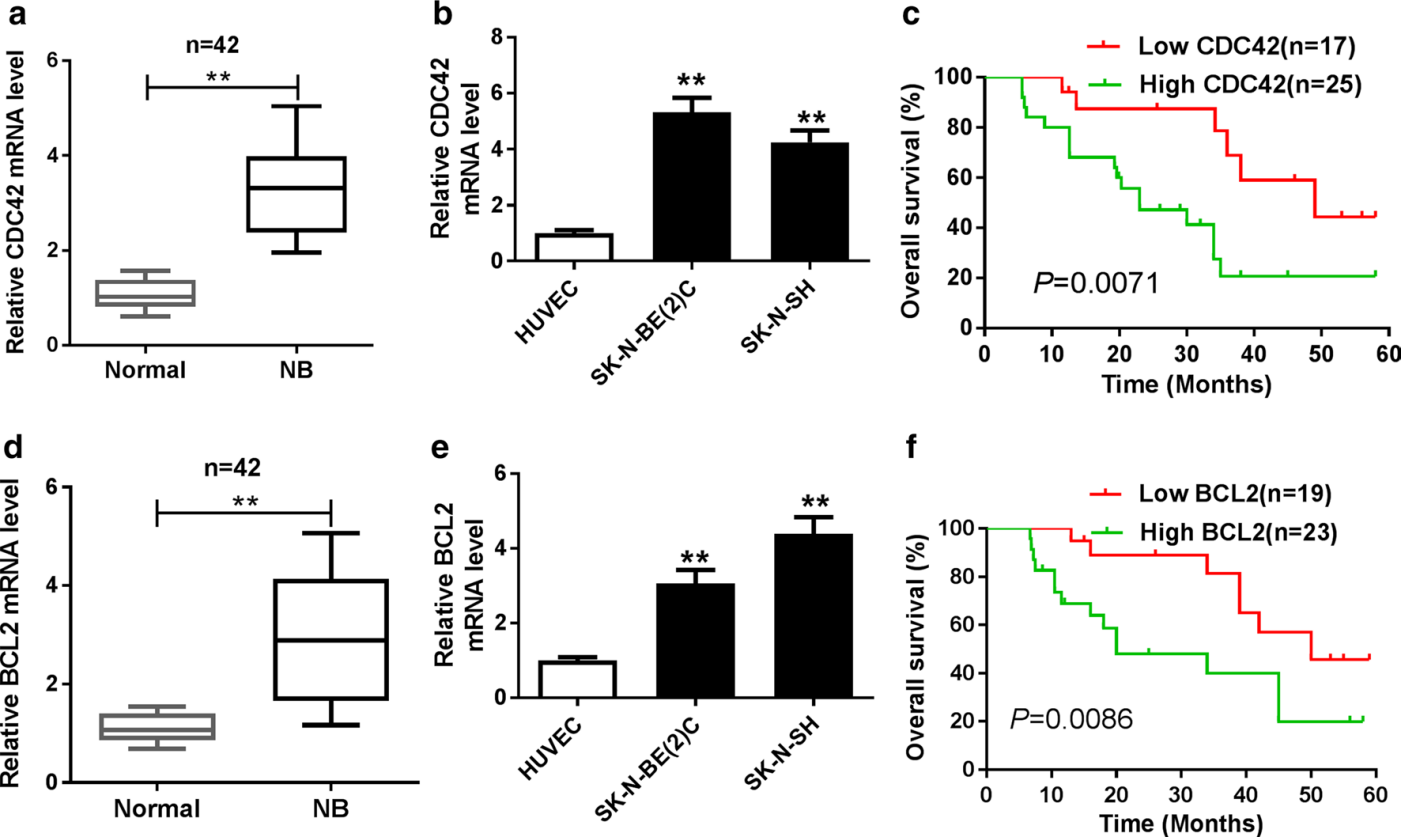
CDC42 and BCL2 mRNA expressions were up-regulated in NB. **a**, **b** The expression of CDC42 mRNA was measured in NB tissues and cells by qRT-PCR. **c** The overall survival was analyzed in patients with high or low expression of CDC42 by Kaplan–Meier method. **d**, **e** The abundance of BCL2 mRNA were examined in NB tissues and cells by qRT-PCR. **f** The overall survival was analyzed in patients with high or low expression of BCL2 by Kaplan–Meier method. **P < 0.01, compared with normal or HUVEC group

To explore the potential roles of CDC42 and BCL2 in NB, their expression levels were measured in NB tissues and cells. Results displayed that the expression of CDC42 mRNA was aberrantly enhanced in NB tissues (n = 42) and cells compared with that in their corresponding control groups (Fig. 5a, b). Moreover, the patients were classified as high CDC42 (n = 25) and low CDC42 expression group (n = 17) according to the mean value of expression level, and high CDC42 expression group showed poor survival (*P *= 0.0071) (Fig. 5c). Meanwhile, the abundance of BCL2 mRNA was also increased in NB tissues (n = 42) and cells (Fig. 5d, e). In addition, patients were classified as high BCL2 (n = 23) and low BCL2 expression (n = 19) according to the mean value of expression level, and high BCL2 expression group showed lower survival (*P *= 0.0086) (Fig. 5f).

### CDC42 is required for miR-149-mediated NB progression

**Fig. 6:**
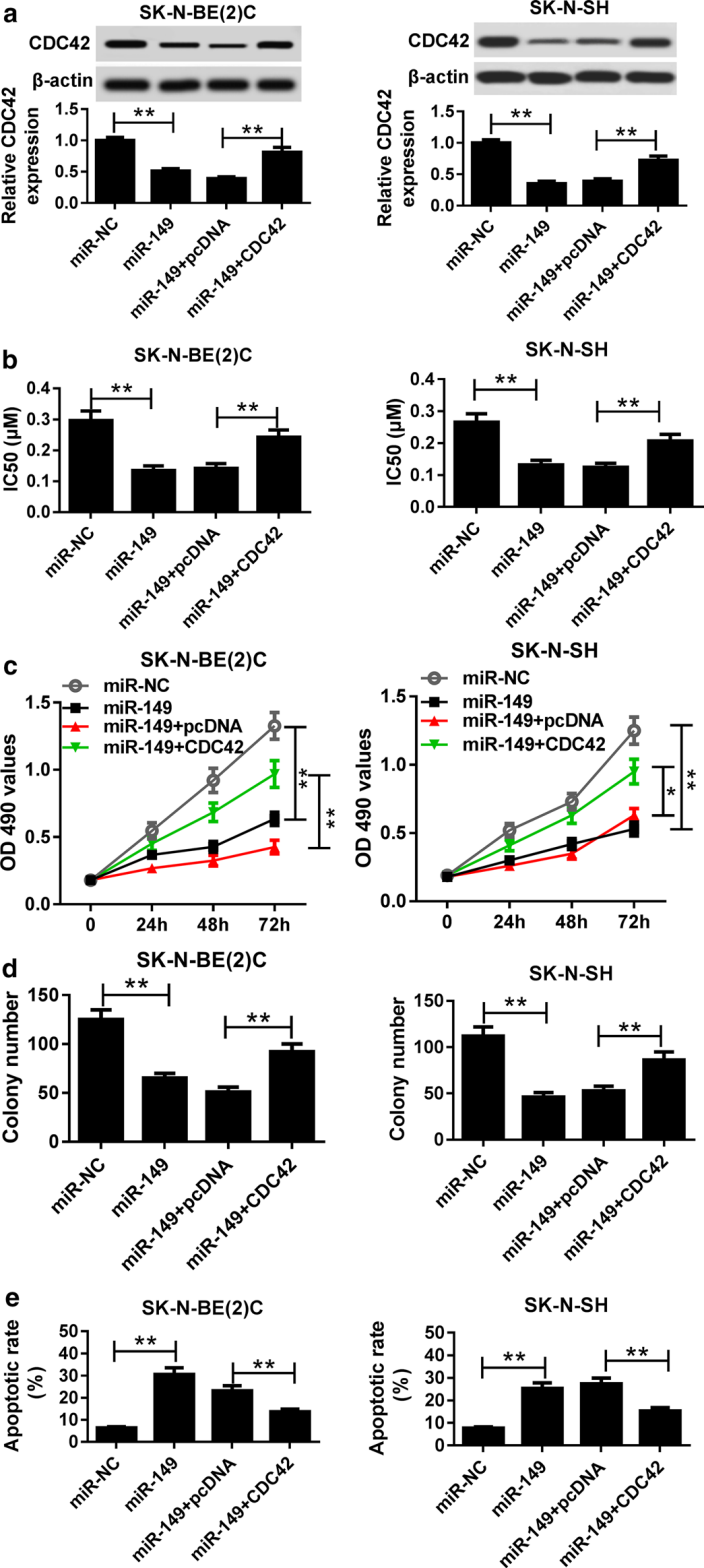
CDC42 was required for miR-149-mediated progression of NB. **a** The expression of CDC42 protein was measured in SK-N-BE(2)C and SK-N-SH cells transfected with miR-NC, miR-149, miR-149 + pcDNA or miR-149 + CDC42 by western blot. The IC50 of Dox (**b**) and cell proliferation (**c**) was analyzed in SK-N-BE(2)C and SK-N-SH cells transfected with miR-NC, miR-149, miR-149 + pcDNA or miR-149 + CDC42 by MTT. **d** Colony formation was measured in SK-N-BE(2)C and SK-N-SH cells transfected with miR-NC, miR-149, miR-149 + pcDNA or miR-149 + CDC42. **e** Cell apoptosis was detected in SK-N-BE(2)C and SK-N-SH cells transfected with miR-NC, miR-149, miR-149 + pcDNA or miR-149 + CDC42 by flow cytometry. *P < 0.05, **P < 0.01, compared with miR-NC or miR-149 + pcDNA group

To investigate whether CDC42 was implicated in miR-149-mediated NB progression, SK-N-BE(2)C and SK-N-SH cells were transfected with miR-NC, miR-149, miR-149 + pcDNA or miR-149 + CDC42. Western blot assay exhibited that overexpression of miR-149 inhibited CDC42 protein level in SK-N-BE(2)C and SK-N-SH cells, whereas introduction of CDC42 expression vector reversed the abundance (Fig. 6a). Moreover, the IC50 value of Dox in SK-N-BE(2)C and SK-N-SH cells was obviously reduced by addition of miR-149, which was attenuated by restoration of CDC42 (Fig. 6b). In addition, up-regulation of CDC42 ablated miR-149-mediated proliferation inhibition in SK-N-BE(2)C and SK-N-SH cells (Fig. 6c). Similarly, overexpression of CDC42 weakened the inhibitory effect of miR-149 on colony formation in SK-N-BE(2)C and SK-N-SH cells (Fig. 6d). Besides, restoration of CDC42 protected against miR-149-induced apoptosis in SK-N-BE(2)C and SK-N-SH cells (Fig. 6e).

### miR-149 mediates NB progression by targeting BCL2

**Fig. 7:**
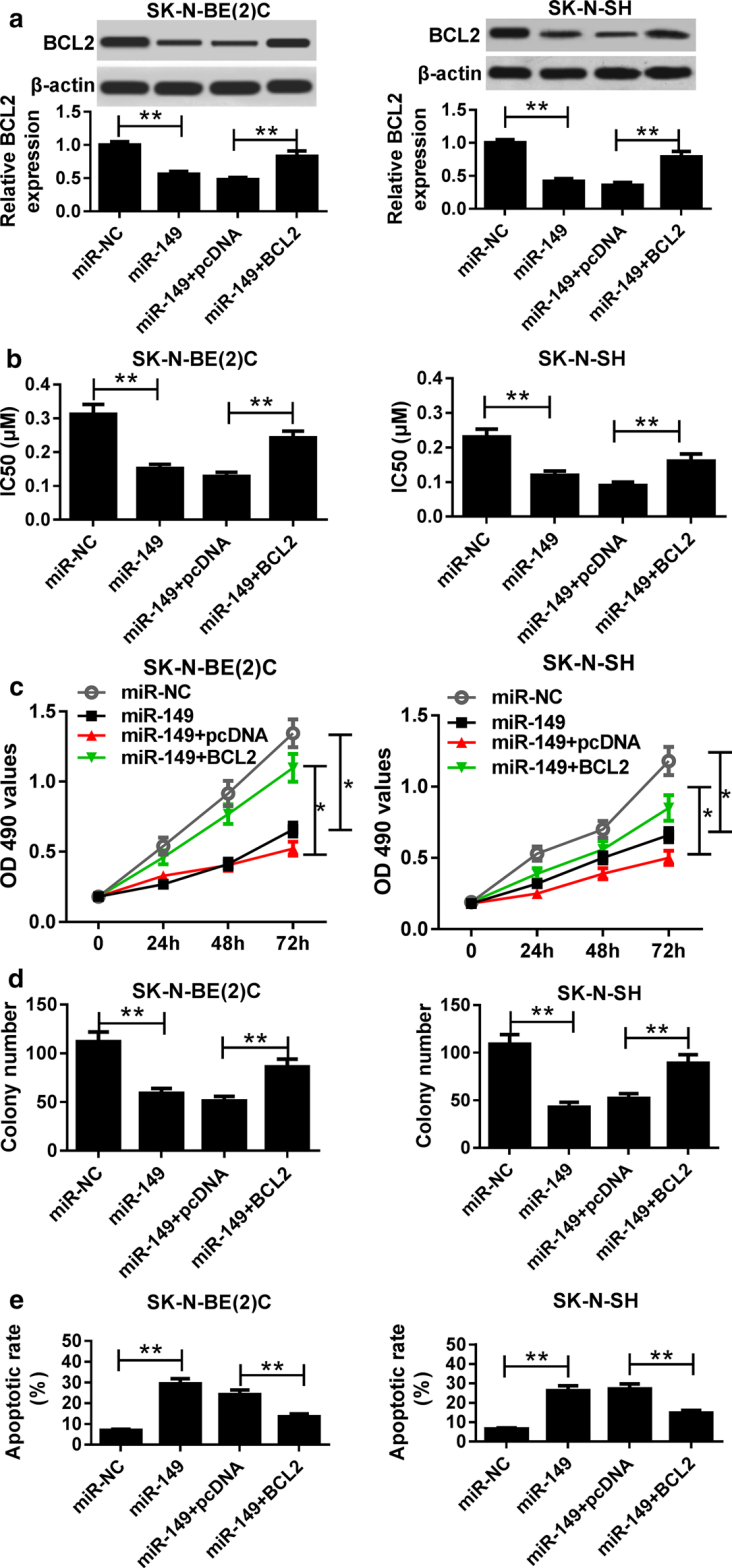
miR-149 regulated NB progression by targeting BCL2. **a** The expression of BCL2 protein was measured in SK-N-BE(2)C and SK-N-SH cells transfected with miR-NC, miR-149, miR-149 + pcDNA or miR-149 + BCL2 by western blot. The IC50 of Dox (**b**), cell proliferation (**c**), colony formation (**d**) and apoptosis (**e**) were analyzed in SK-N-BE(2)C and SK-N-SH cells transfected with miR-NC, miR-149, miR-149 + pcDNA or miR-149 + BCL2 by MTT or flow cytometry, respectively. *P < 0.05, **P < 0.01, compared with miR-NC or miR-149 + pcDNA group

To explore whether BCL2 was involved in miR-149-mediated NB progression, SK-N-BE(2)C and SK-N-SH cells were transfected with miR-NC, miR-149, miR-149 + pcDNA or miR-149 + BCL2. As a result, the expression of BCL2 protein was drastically decreased by transfection of miR-149 in SK-N-BE(2)C and SK-N-SH cells, which was reversed by introduction of BCL2 expression vector (Fig. 7a). Furthermore, overexpression of miR-149 led to a great loss of IC50 value of Dox in SK-N-BE(2)C and SK-N-SH cells, which was counteracted by restoration of BCL2 (Fig. 7b). Additionally, overexpression of BCL2 ablated miR-149-mediated inhibition of proliferation of SK-N-BE(2)C and SK-N-SH cells (Fig. 7c). Similarly, increase of BCL2 protected colony formation from miR-149 in SK-N-BE(2)C and SK-N-SH cells (Fig. 7d). Besides, restoration of BCL2 ameliorated miR-149-induced apoptosis in SK-N-BE(2)C and SK-N-SH cells (Fig. 7e).

## Discussion

miRNAs have been reported to have pivotal roles in regulating cell phenotypes, differentiation, tumorigenicity and chemoresistance in NB [24, 25]. Here we found that miR-149 expression was declined in NB and associated with low survival of patients, which is also consistent with former work [12]. However, the role and mechanism allows miR-149 participating in NB remains poorly understood. In this study, we investigated the role of miR-149 in NB development and chemosensitivity, and we were the first to provide miR-149 enhanced Dox sensitivity. Moreover, this study first validated the target interaction between miR-149 and CDC42/BCL2 in NB.

Previous study indicated that miR-149 inhibited cell proliferation in NB cells [12]. Moreover, miR-149 promoted cell apoptosis by regulating threonine-protein kinase (Akt1) and E2 factor 1 (E2F1) in NB cells [26]. Similarly, we also found that miR-149 suppressed cell proliferation and induced apoptosis in NB cells. Moreover, chemoresistance was a challenge in therapy of NB. In this study, we found that up-regulation of miR-149 enhanced Dox sensitivity in NB cells, indicating that miR-149 might act as a sensitizer in chemotherapy, which was also in agreement with that in ovarian cancer and breast cancer [11, 27]. Collectively, our study indicated that miR-149 might play an anti-cancer role in NB by inhibiting NB development and increasing chemosensitivity. However, the mechanism by which miR-149 was implicated in progression and chemosensitivity of NB cells remains dismal.

The network of miRNA-mRNA interaction played essential roles in tumorigenesis of NB [28]. Previous efforts have identified various targets of miR-149 in different conditions. For example, miR-149 regulated cell proliferation and apoptosis by targeting G-protein-coupled receptor (GPCR)-kinase interacting protein-1 (GIT1) in cervical cancer [9]. Moreover, miR-149 contributed to myocardial differentiation by regulating disabled homolog 2 (Dab2) in mouse bone marrow stem cells [29]. In addition, miR-149 enhanced sensitivity of colorectal cancer to radiotherapy by targeting human epididymis protein 4 (HE4) [30]. However, little is known about the interaction between miR-149 and CDC42 as well as BCL2. This study was the first to identify the interaction in NB cells by luciferase activity and RIP assays.

CDC42 has been reported to contribute to cancer progression by participating in cell cycle to induce cell proliferation in various cancers [31]. Moreover, inhibition of CDC42 was regarded as a therapeutic target in cancers [32]. Notably, previous study showed that CDC42 was enhanced and its knockdown inhibited cell proliferation and colony formation but induced cell death in NB [33]. Similarly, we also found high expression of CDC42 in NB and its overexpression reversed miR-149-mediated inhibition of proliferation as well as colony formation and promotion of apoptosis. In addition, this study revealed that addition of CDC42 decreased Dox sensitivity in NB cells, which is also in agreement with former work that suggested CDC42 enhanced chemoresistance in pancreatic cancer [34].

BCL2, as an anti-apoptotic factor, was suggested to inhibit neuronal cell death [35]. In the present study, BCL2 expression was enhanced in NB and its overexpression abated miR-149-mediated inhibition of cell proliferation. This is also in agreement with previous study suggested that BCL2 inhibition suppressed tumor growth in NB [36]. Moreover, BCL2 could inhibit sensitivity of NB cells to cisplatin [37]. We also uncovered that BCL2 suppressed sensitivity of NB cells to Dox. Pre-clinical xenograft experiment is responsible for better understanding the role and mechanism of miR-149 in NB. Hence, in vivo study with animal model is needed in future.

## Conclusion

In conclusion, miR-149 level was reduced and its overexpression inhibited cell proliferation but promoted cell apoptosis and Dox chemosensitivity in NB, possibly by targeting CDC42 and BCL2, providing new insight into therapeutic target of miR-149 for NB treatment.

## Supplementary information

**Additional file 1: Figure S1.** The expression of predicted targets of miR-149 in SK-N-SH cells. The expression levels of 8 predicted targets of miR-149 were detected in SK-N-SH cells and HUVEC cells. *P < 0.05.

## Data Availability

All data generated or analyzed during this study are included in this published article.

## References

[1] Cheung N, Dyer M (2013). Neuroblastoma: developmental biology, cancer genomics and immunotherapy. Nat Rev Cancer.

[2] Pinto N, Applebaum M, Volchenboum S, Matthay K, London W, Ambros P, Nakagawara A, Berthold F, Schleiermacher G, Park J (2015). Advances in risk classification and treatment strategies for neuroblastoma. J Clin Oncol.

[3] Ridinger J, Koeneke E, Kolbinger F, Koerholz K, Mahboobi S, Hellweg L, Gunkel N, Miller A, Peterziel H, Schmezer P (2018). Dual role of HDAC10 in lysosomal exocytosis and DNA repair promotes neuroblastoma chemoresistance. Sci Rep.

[4] Galardi A, Colletti M, Businaro P, Quintarelli C, Locatelli F, Di Giannatale A (2018). MicroRNAs in neuroblastoma: biomarkers with therapeutic potential. Curr Med Chem.

[5] Buhagiar A, Ayers D (2015). Chemoresistance, cancer stem cells, and miRNA influences: the case for neuroblastoma. Anal Cell Pathol.

[6] Zhi Y, Zhou H, Mubalake A, Chen Y, Zhang B, Zhang K, Chu X, Wang R (2018). Regulation and functions of microRNA-149 in human cancers. Cell Prolif.

[7] Shi X, Wang X, Hua Y (2018). LncRNA GACAT1 promotes gastric cancer cell growth, invasion and migration by regulating MiR-149-mediated of ZBTB2 and SP1. J Cancer..

[8] Chayeb V, Mahjoub S, Zitouni H, Jrah-Harzallah H, Zouari K, Letaief R, Mahjoub T (2018). Contribution of microRNA-149, microRNA-146a, and microRNA-196a2 SNPs in colorectal cancer risk and clinicopathological features in Tunisia. Gene.

[9] Qian B, Zhao L, Wang X, Xu J, Teng F, Gao L, Shen R (2018). miR-149 regulates the proliferation and apoptosis of cervical cancer cells by targeting GIT1. Biomed Pharmacother.

[10] Li Y, Ju K, Wang W, Liu Z, Xie H, Jiang Y, Jiang G, Lu J, Dong Z, Tang F (2018). Dinitrosopiperazine-decreased PKP3 through upregulating miR-149 participates in nasopharyngeal carcinoma metastasis. Mol Carcinog.

[11] Sun L, Zhai R, Zhang L, Zhao S (2018). MicroRNA-149 suppresses the proliferation and increases the sensitivity of ovarian cancer cells to cisplatin by targeting X-linked inhibitor of apoptosis. Oncol Lett.

[12] Xu Y, Chen X, Lin L, Chen H, Yu S, Li D (2017). MicroRNA-149 is associated with clinical outcome in human neuroblastoma and modulates cancer cell proliferation through Rap1 independent of MYCN amplification. Biochimie.

[13] Maldonado M, Dharmawardhane S (2018). Targeting Rac and Cdc42 GTPases in cancer. Cancer Res.

[14] Shi L, Xiao R, Wang M, Zhang M, Weng N, Zhao X, Zheng X, Wang H, Mai S (2018). MicroRNA-342-3p suppresses proliferation and invasion of nasopharyngeal carcinoma cells by directly targeting Cdc42. Oncol Rep.

[15] Li X, Jiang M, Chen D, Xu B, Wang R, Chu Y, Wang W, Zhou L, Lei Z, Nie Y (2018). miR-148b-3p inhibits gastric cancer metastasis by inhibiting the Dock6/Rac1/Cdc42 axis. J Exp Clin Cancer Res.

[16] Shi C, Ren L, Sun C, Yu L, Bian X, Zhou X, Wen Y, Hua D, Zhao S, Luo W (2017). miR-29a/b/c function as invasion suppressors for gliomas by targeting CDC42 and predict the prognosis of patients. Br J Cancer.

[17] Xu T, Xie H, Li Y, Xia Y, Chen Y, Xu L, Wang L, Zhao B (2017). CDC42 expression is altered by dioxin exposure and mediated by multilevel regulations via AhR in human neuroblastoma cells. Sci Rep.

[18] Pfeffer C, Singh A (2018). Apoptosis: a target for anticancer therapy. Int J Mol Sci.

[19] Radha G, Raghavan S (2017). BCL2: a promising cancer therapeutic target. Biochim Biophys Acta Rev Cancer..

[20] Cole K, Attiyeh E, Mosse Y, Laquaglia M, Diskin S, Brodeur G, Maris J (2008). A functional screen identifies miR-34a as a candidate neuroblastoma tumor suppressor gene. Mol Cancer Res.

[21] Livak KJ, Schmittgen TD (2001). Analysis of relative gene expression data using real-time quantitative PCR and the 2^−ΔΔCT^ method. Methods.

[22] Ren W, Wu S, Wu Y, Liu T, Zhao X, Li Y (2019). MicroRNA-196a/-196b regulate the progression of hepatocellular carcinoma through modulating the JAK/STAT pathway via targeting SOCS2. Cell Death Dis..

[23] Gao R, Zhang N, Yang J, Zhu Y, Zhang Z, Wang J, Xu X, Li Z, Liu X, Li Z, Li J, Kong C, Bi J (2019). Long non-coding RNA ZEB1-AS1 regulates miR-200b/FSCN1 signaling and enhances migration and invasion induced by TGF-beta1 in bladder cancer cells. J Exp Clin Cancer Res..

[24] Samaraweera L, Grandinetti K, Huang R, Spengler B, Ross R (2014). MicroRNAs define distinct human neuroblastoma cell phenotypes and regulate their differentiation and tumorigenicity. BMC Cancer.

[25] Ayers D, Mestdagh P, Van Maerken T, Vandesompele J (2015). Identification of miRNAs contributing to neuroblastoma chemoresistance. Comput Struct Biotechnol J..

[26] Lin R, Lin Y, Yu A (2010). miR-149* induces apoptosis by inhibiting Akt1 and E2F1 in human cancer cells. Mol Carcinog.

[27] He D, Gu X, Li Y, Jiang L, Jin J, Ma X (2014). Methylation-regulated miR-149 modulates chemoresistance by targeting GlcNAc N-deacetylase/N-sulfotransferase-1 in human breast cancer. FEBS J.

[28] Ooi C, Carter D, Liu B, Mayoh C, Beckers A, Lalwani A, Nagy Z, De Brouwer S, Decaesteker B, Hung T (2018). Network modeling of microRNA-mRNA interactions in neuroblastoma tumorigenesis identifies miR-204 as a direct inhibitor of MYCN. Cancer Res.

[29] Lu M, Xu L, Wang M, Guo T, Luo F, Su N, Yi S, Chen T (2018). miR-149 promotes the myocardial differentiation of mouse bone marrow stem cells by targeting Dab2. Mol Med Rep..

[30] Shi L, Guo H, Su Y, Zheng Z, Liu J, Lai S (2018). MicroRNA-149 sensitizes colorectal cancer to radiotherapy by downregulating human epididymis protein 4. Am J Cancer Res..

[31] Xiao X, Lv L, Duan J, Wu Y, He S, Hu Z, Xiong L (2018). Regulating Cdc42 and its signaling pathways in cancer: small molecules and microRNA as new treatment candidates. Molecules.

[32] Aguilar B, Zhou H, Lu Q (2017). Cdc42 signaling pathway inhibition as a therapeutic target in Ras-related cancers. Curr Med Chem.

[33] Lee S, Craig B, Romain C, Qiao J, Chung D (2014). Silencing of CDC42 inhibits neuroblastoma cell proliferation and transformation. Cancer Lett.

[34] Yang D, Tang Y, Fu H, Xu J, Hu Z, Zhang Y, Cai Q (2018). Integrin β1 promotes gemcitabine resistance in pancreatic cancer through Cdc42 activation of PI3K p110β signaling. Biochem Biophys Res Commun.

[35] Yadav S, Pandey A, Shukla A, Talwelkar S, Kumar A, Pant A, Parmar D (2011). miR-497 and miR-302b regulate ethanol-induced neuronal cell death through BCL2 protein and cyclin D2. J Biol Chem.

[36] Lamers F, Schild L, den Hartog I, Ebus M, Westerhout E, Ora I, Koster J, Versteeg R, Caron H, Molenaar J (2012). Targeted BCL2 inhibition effectively inhibits neuroblastoma tumour growth. Eur J Cancer.

[37] Ryan J, Tivnan A, Fay J, Bryan K, Meehan M, Creevey L, Lynch J, Bray I, O’Meara A, Tracey L (2012). MicroRNA-204 increases sensitivity of neuroblastoma cells to cisplatin and is associated with a favourable clinical outcome. Br J Cancer.

