# Supplementary figures and images for: miR-149 inhibits cell proliferation and enhances chemosensitivity by targeting CDC42 and BCL2 in neuroblastoma
Source: Cancer Cell Int. 2019 Dec 27;19:357. doi: 10.1186/s12935-019-1082-9 (PMC6935209; doi:10.1186/s12935-019-1082-9)

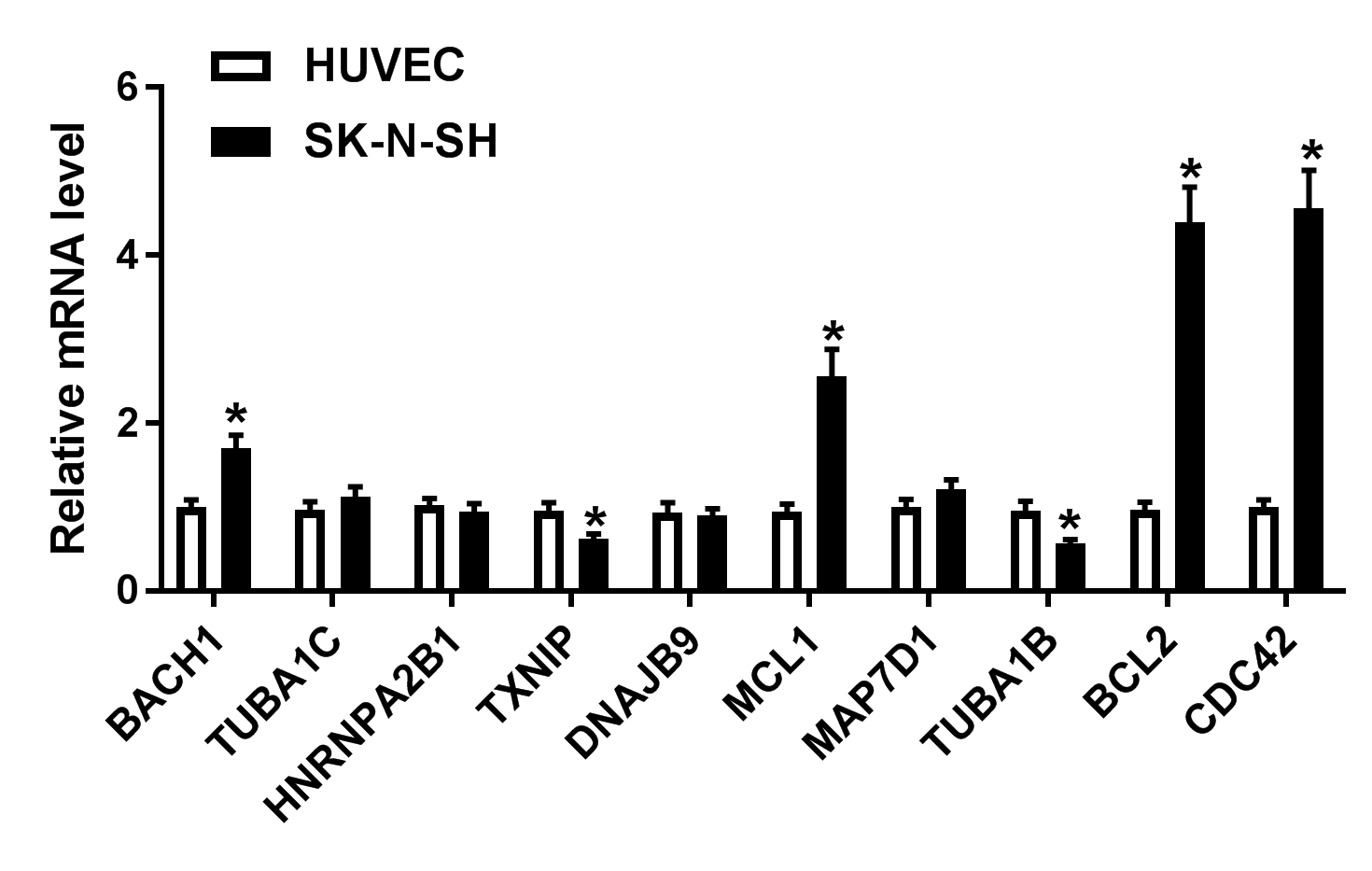

Supplement: Supplementary file 1 — Additional file 1: Figure S1. The expression of predicted targets of miR-149 in SK-N-SH cells. The expression levels of 8 predicted targets of miR-149 were detected in SK-N-SH cells and HUVEC cells. *P < 0.05. [file 12935_2019_1082_MOESM1_ESM.tif]
